# Trends in peripheral nerve injury research: a bibliometric analysis focused on molecular mechanisms

**DOI:** 10.3389/fneur.2026.1771375

**Published:** 2026-03-06

**Authors:** Guohua Jiang, Renkun Huang, Yuchang Gui, Guiyang Jiang, Kewen Wang, Yijun Liu, Jianwen Xu

**Affiliations:** 1 Department of Rehabilitation Medicine, The First Affiliated Hospital of Guangxi Medical University, Nanning, Guangxi, China; 2 Department of Foot and Ankle Surgery, Center for Orthopedic Surgery, The Third Affiliated Hospital of Southern Medical University, Guangzhou, China

**Keywords:** bibliometric analysis, molecular mechanisms, nerve regeneration, peripheral nerve injury, visualization analysis

## Abstract

**Objective:**

Peripheral nerve injury (PNI) remains a significant clinical challenge due to its limited recovery outcomes. Understanding the underlying molecular mechanisms is crucial for improving therapeutic efficacy. This study aims to explore the current status, research hotspots, and development trends of molecular mechanism studies in PNI through bibliometric analysis, providing valuable insights for the development of more effective treatment strategies.

**Methods:**

Relevant literature on PNI and its molecular mechanisms published from January 1, 2005, to November 22, 2025, was retrieved from the Web of Science, PubMed, and Scopus databases. CiteSpace and VOSviewer software were utilized to analyze research hotspots and trends, generating visual maps of countries, institutions, authors, journals, keywords, and references.

**Results:**

A total of 1,799 publications were analyzed, revealing a steady annual increase in PNI-related research, with a significant surge after 2016. China emerged as the leading contributor, followed by the United States and Japan. Nantong University and Sun Yat-sen University were identified as the major contributing institutions, with Gu Xiaosong being one of the most influential authors. Key journals in the field include *Neural Regeneration Research* and *Molecular Pain*. Research hotspots include Schwann cells, neuropathic pain, inflammatory responses, non-coding RNAs, and molecular signaling pathways. Notably, advances in bioinformatics technologies, such as high-throughput genomics, proteomics, transcriptomics, and single-cell sequencing, have significantly propelled the research on PNI molecular mechanisms. Future research is likely to focus on the application of precision medicine and gene editing technologies to enhance PNI treatment outcomes.

**Conclusion:**

This bibliometric analysis provides a comprehensive overview of the current status and trends in PNI molecular mechanism research, revealing key research areas and future directions. The advancement of bioinformatics technologies is expected to drive the development of future therapeutic strategies, offering new avenues for nerve regeneration and functional recovery.

## 1 Introduction

Peripheral nerve injury (PNI) is a common clinical condition caused by trauma, compression, traction, or other pathological factors that lead to damage of the structural and functional integrity of peripheral nerves. PNI often results in symptoms such as sensory loss, motor dysfunction, and chronic pain, severely impacting the patient’s quality of life (1). Unlike the central nervous system, peripheral nerves possess a certain degree of regenerative capacity. However, this regeneration process is generally slow and rarely leads to complete functional recovery, especially in cases of severe injury (2). Although current therapeutic approaches, such as surgical repair and nerve transplantation, have shown some success in promoting nerve recovery, they often fail to achieve optimal clinical outcomes (3).

At the molecular level, the repair of peripheral nerve injury involves dynamic changes in multiple cell types, including Schwann cell dedifferentiation, proliferation, and remyelination, the recruitment of inflammatory cells (such as macrophages), and the regulation of axonal regeneration mechanisms. These processes are controlled by complex molecular signaling pathways, such as Wallerian degeneration, neurotrophic factor signaling, and the activation of transcription factors like c-Jun (4–6). Therefore, a deeper understanding of these molecular mechanisms is crucial for revealing the pathological processes underlying PNI and developing more effective regenerative therapies.

Bibliometric analysis, a research tool that uses mathematical and statistical methods to quantitatively analyze scientific outputs, provides valuable insights into the development trajectory of a particular field, identifying key institutions, influential authors, core journals, research hotspots, and future trends (7). In fields such as neuroscience and regenerative medicine, bibliometric analysis has become an essential method for understanding research trends and evaluating scientific achievements (8). With the rapid advancement of basic biology and high-throughput technologies, research on the molecular mechanisms of PNI has significantly increased, making it difficult for traditional reviews to fully capture the scope of this interdisciplinary field. Bibliometric methods offer a comprehensive macro perspective, systematically summarizing existing research, identifying trends and hotspots, and assisting in predicting future research directions.

This study aims to utilize bibliometric tools, such as CiteSpace and VOSviewer, to visually analyze the molecular mechanism research on PNI published in the past two decades. By doing so, we seek to explore the current state of research and identify key research hotspots in the field, providing valuable reference and theoretical support for future studies.

## 2 Materials and methods

### 2.1 Data sources

To ensure the comprehensiveness, authority, and diversity of the data, this study collected relevant literature from three internationally recognized databases: Web of Science Core Collection (WoSCC), PubMed, and Scopus. WoSCC is one of the most authoritative databases for academic publications and citation analysis, covering approximately 34,000 high-quality journals across multiple disciplines. The high-quality citation data and standardized metadata it provides are compatible with mainstream bibliometric software, making it a core tool for analyzing academic trends, collaboration networks, and research hotspots (9). Scopus, launched by Elsevier in 2004, is a comprehensive abstract and citation database that includes a large number of high-quality publications across various disciplines, complementing WoSCC’s literature coverage (10). PubMed focuses on biomedical and life sciences literature, capturing publications that are not fully covered by the other two databases, thus broadening the scope of the literature search (11).

All literature retrievals were conducted on the same day to avoid potential bias due to daily database updates. The time frame for the search was set from January 1, 2005, to November 22, 2025. The bibliometric analysis strictly followed the Preferred Reporting Items for Systematic Reviews and Meta-Analyses 2020 (PRISMA 2020) guidelines for data extraction and analysis. Inclusion criteria: (1) Document types: original research articles and review articles; (2) Language: only English-language publications. Exclusion criteria: (1) Non-English publications; (2) Document types: preprints, conference papers, conference abstracts, book chapters, letters, and retracted articles; (3) Publications unrelated to the molecular mechanisms of PNI.

### 2.2 Search strategy and screening process

The search formula used in Web of Science was as follows: TS=(“peripheral nerve injury” OR “peripheral nerve lesion” OR “peripheral nerve trauma” OR “PNI”) AND TS=(“molecular mechanism” OR “molecular pathway” OR “signaling pathway” OR “signal transduction”) AND DT=(Article OR Review) AND LA=(ENGLISH). Publication Years: 2005-2025.

The search formula used in Scopus was: TITLE-ABS-KEY (“peripheral nerve injury” OR “peripheral nerve lesion” OR “peripheral nerve trauma” OR “PNI”) AND TITLE-ABS-KEY (“molecular mechanism” OR “molecular pathway” OR “signaling pathway” OR “signal transduction”) AND DOCTYPE (ar OR re) AND LANGUAGE (English) AND [PUBDATETXT (01 January 2005) AND PUBDATETXT (22 November 2025)].

The search formula used in PubMed was: (“peripheral nerve injury”[MeSH Terms] OR “peripheral nerve lesion”[Title/Abstract] OR “peripheral nerve trauma”[Title/Abstract] OR “PNI”[Title/Abstract]) AND (“molecular mechanism”[Title/Abstract] OR “molecular pathway”[Title/Abstract] OR “signaling pathway”[Title/Abstract] OR “signal transduction”[Title/Abstract]) AND ((2005/1/1:2025/11/22[pdat]) AND (English[Filter])). Article types include research articles, studies, and reviews.

**Figure 1:**
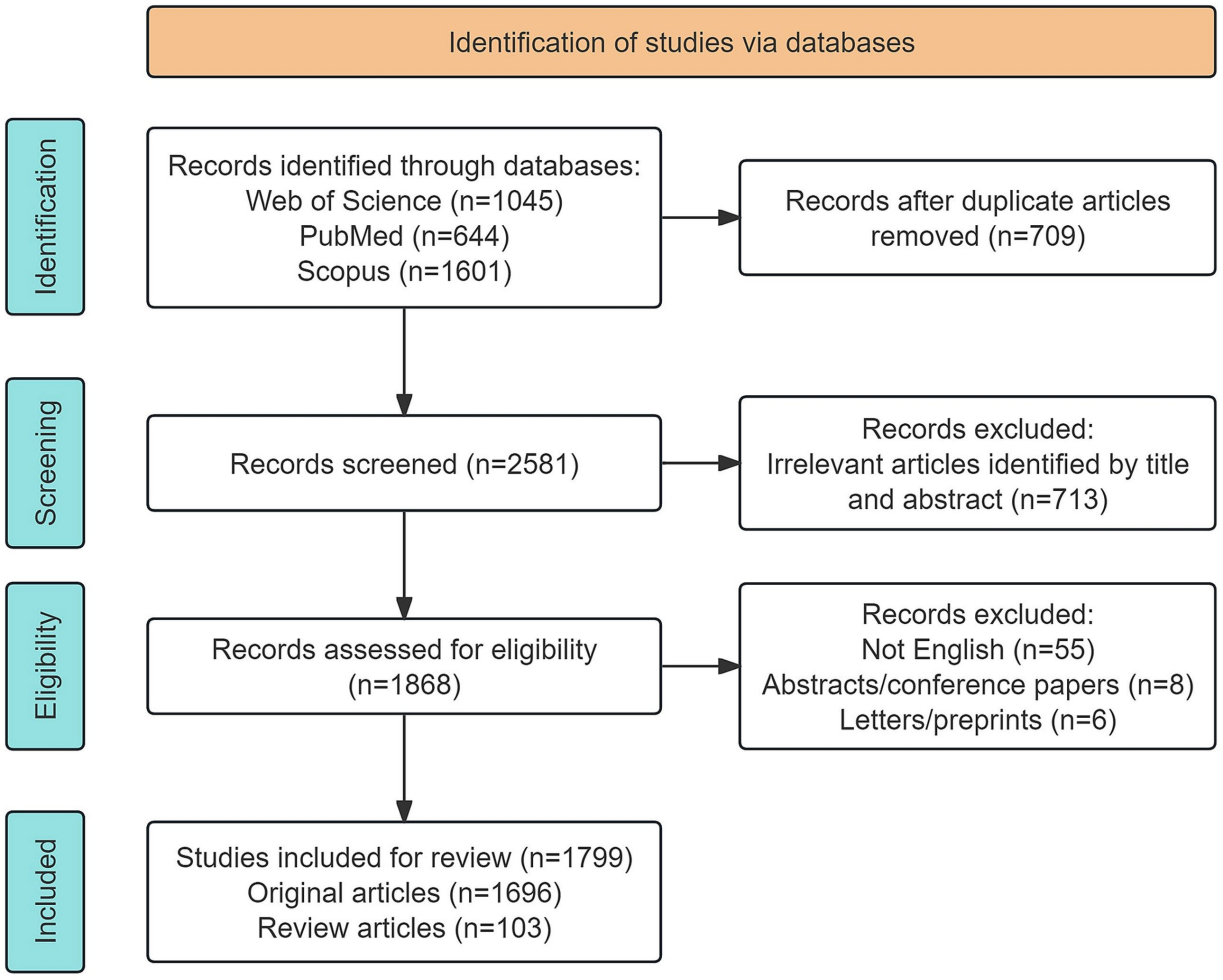
Flowchart of the literature screening process.

Subsequently, two independent researchers (Guohua Jiang and Renkun Huang) conducted a two-stage screening process: first, screening based on titles and abstracts to exclude obviously irrelevant literature, and second, full-text screening to further verify relevance to the research topic. Disagreements between the two researchers were resolved through consultation with a third senior researcher (Yuchang Gui), who made the final decision. A flow diagram of the entire literature screening process is shown in Figure 1.

### 2.3 Data standardization

This study utilized VOSviewer software to identify countries/regions and keywords in the literature, followed by data cleaning and standardization through the merging and modification functions of the “lexicon file.” The specific operations were as follows:

(1) Standardization of country/region names:

The internationally recognized names were used for countries, e.g., “Peoples R China” was standardized to “China,” “United States” was simplified to “United States,” and “England” was changed to “United Kingdom.”

The relationship between regions and their respective countries was clarified. For example, Taiwan, Hong Kong, and Macau were classified under “China,” and Scotland and Wales were grouped under the “United Kingdom” to ensure consistent geographical information.

(2) Standardization of keywords:

Merging synonymous keywords: Keywords with the same meaning were unified, such as “axonal regeneration” and “axon regeneration” being merged into “axon regeneration,” and “peripheral-nerve injury” and “peripheral nerve injury” being unified as “peripheral nerve injury.”

Standardizing different expressions: Keywords with different spelling formats but the same core meaning were standardized, such as “dorsal-root ganglion” to “dorsal root ganglion” and “gene-expression” to “gene expression.”

Singular/plural form standardization: Plural forms were standardized to singular forms, such as “animals” to “animal,” “axons” to “axon,” and “schwann cells” to “schwann cell.”

All merged and modified country/region names and keywords are documented in Supplementary Table S1.

### 2.4 Visualization tools and analysis

This study employed various specialized software tools for bibliometric analysis and data visualization of the molecular mechanisms of peripheral nerve injury: VOSviewer (version 1.6.20) was used to construct visual knowledge maps of collaboration networks among countries/regions, institutions, authors, journals, and co-occurrence networks of keywords. CiteSpace (version 6.4. R1) employed time slicing technology to create temporal network models, generate journal dual-map overlays, co-citation cluster timelines, and conduct keyword and reference burst detection. Pajek64 (version 6.01) and SCImago Graphica (version 1.0.53) were used to optimize cluster views for enhanced visualization. Rstudio (Bibliometrix 5.0) was used to analyze the collaboration rates between different countries and generate keyword quadrant plots. Microsoft Office Excel 2019 was used for data summarization, table construction, and the creation of publication trend graphs.

## 3 Results

### 3.1 Annual publication trend

**Figure 2:**
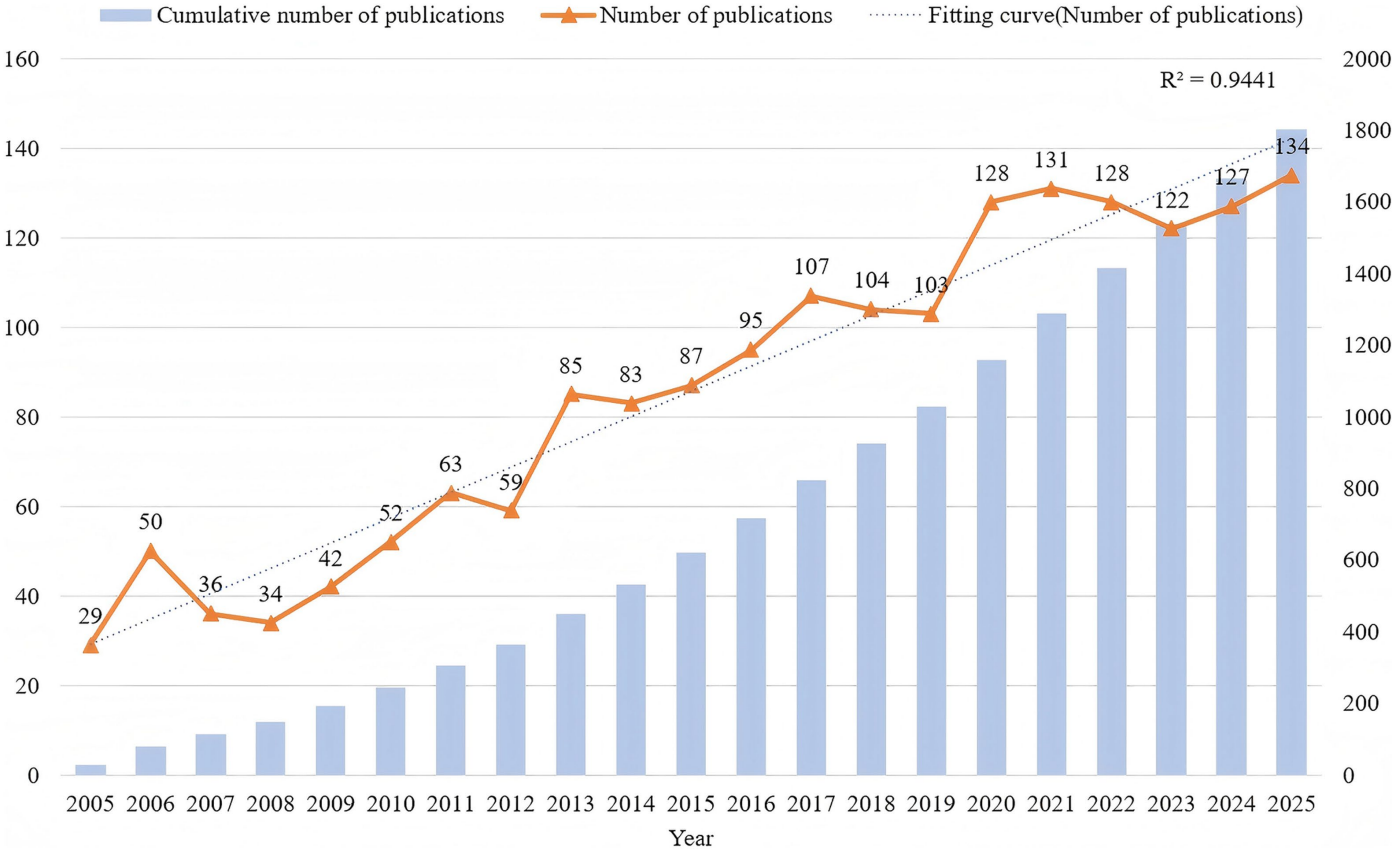
Annual publication volume and cumulative publication trends in research on the molecular mechanisms of peripheral nerve injury. The orange line represents the annual publication volume, the blue bars represent cumulative publications, and the blue dashed line represents the fitted publication trend curve.

A total of 1,799 publications were identified from the literature search and screening for the period 2005 to 2025, including 1,409 original articles and 390 reviews. The annual publication trend and cumulative number of publications are shown in Figure 2. From 2005 to 2015, the number of publications showed steady growth. The annual output started at 29 papers in 2005, with slight fluctuations in the early years (e.g., only 34 papers in 2008), but steadily increased to 87 papers by 2015. After 2016, the field entered a phase of rapid growth, with a significant rise in annual publications: reaching 128 papers in 2020, and further increasing to 131 papers in 2021. Although there was a slight decline in 2023 (122 papers), the overall trend remained high, with projections of 127 papers in 2024 and 134 papers in 2025. This temporary downturn between 2021 and 2023 is likely attributable to the prolonged impact of the COVID-19 pandemic, which disrupted laboratory-based research activities, scientific collaboration, and the processes of manuscript submission and peer review, thereby transiently suppressing research productivity. The fitted annual publication curve shows a coefficient of determination (R^2^) of 0.9441, indicating a strong linear growth trend, reflecting the sustained increase in research on molecular mechanisms of PNI. Meanwhile, the cumulative number of publications (represented by the blue bars) continued to rise, surpassing 1,750 papers by 2025, further confirming the growing academic contribution in this research area.

### 3.2 Analysis of countries and institutions

**Figure 3:**
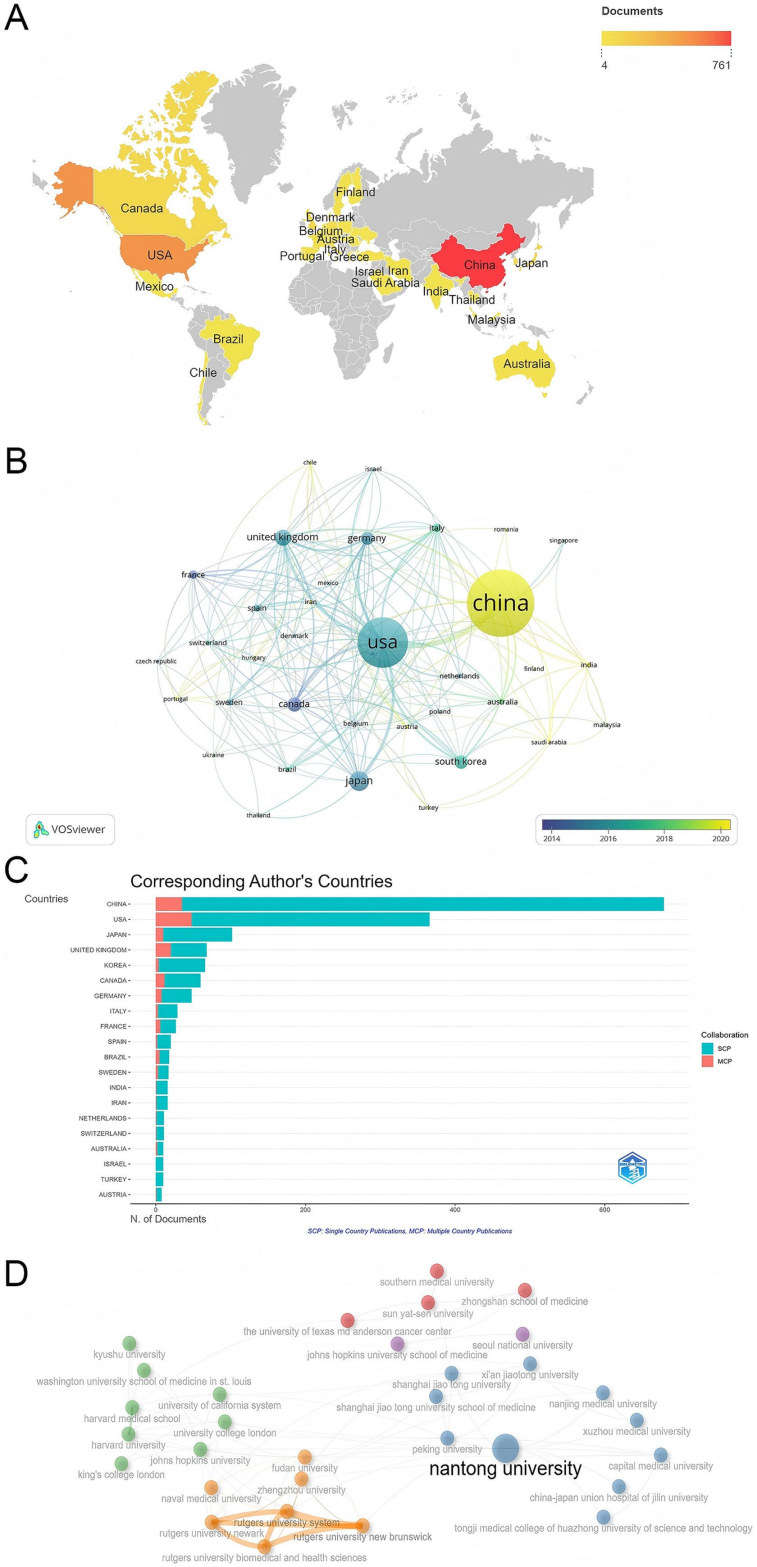
Visualization of countries and institutions involved in PNI molecular mechanism research. **(A)** Global geographical distribution map generated by VOSviewer combined with Scimago Graphica. **(B)** Country collaboration network generated by VOSviewer, with nodes representing countries. Node size correlates with publication volume, and line thickness indicates the strength of collaboration. **(C)** Distribution of independent publications (SCP) and multi-country collaborative publications (MCP) for major countries; the x-axis represents publication count, with different colored bars representing SCP (blue) and MCP (orange). **(D)** Institutional collaboration network generated by VOSviewer, where nodes represent research institutions. Node size is proportional to publication volume, and lines represent collaboration relationships, with line thickness indicating the intensity of collaboration.

Between 2005 and 2025, a total of 166 countries participated in research related to the molecular mechanisms of peripheral nerve injury. The geographical distribution of research output is shown in Figure 3A, indicating a global research trend, with a high concentration in Asia, North America, and Europe. Among the top 10 countries by publication output, China ranked first with 679 papers, accounting for 37.7% of the total publications, far exceeding other countries. This dominance may be closely related to China’s large population base and the growing number of patients affected by peripheral nerve injury, whereby increasing clinical demand has further stimulated basic research activity and the generation of related research outputs in this field. The United States followed with 366 papers (20.3%), while Japan (102 papers, 5.7%), the United Kingdom (68 papers, 3.8%), and South Korea (66 papers, 3.7%) ranked third to fifth. These five countries accounted for more than 70% of the total output, constituting the core contributors to the field. The international collaboration network, shown in Figure 3B, indicates that a broad network of global cooperation has been established. In the figure, nodes represent countries, with node size proportional to publication output, and line thickness indicating the strength of collaboration. The most frequent collaborations were between China and the United States, with both countries forming the core of international collaboration. According to the collaboration type statistics (Figure 3C; Table 1), the United Kingdom had the highest proportion of multi-country collaborative papers (MCPs) at 29.4%, followed by France (22.2%) and Canada (20.0%), reflecting a strong willingness for international collaboration in some European and North American countries. The proportion of MCPs for the United States was 13.1%, contributing to 48 papers, while China produced 35 MCPs, but these accounted for only 5.2% of its total publications, suggesting significant room for expanding international collaboration.

**Table 1:** Collaboration type statistics of major countries.

Country	Publications	SCP	MCP	MCP (%)
China	679	644	35	5.2
United States	366	318	48	13.1
Japan	102	92	10	9.8
United Kingdom	68	48	20	29.4
Korea	66	62	4	6.1
Canada	60	48	12	20.0
Germany	48	40	8	16.7
Italy	29	26	3	10.3
France	27	21	6	22.2
Spain	20	18	2	10.0

SCP, Single-Country Publication; MCP, Multi-Country Publication.

VOSviewer analysis revealed that 1,644 research institutions contributed to the publications in this field, with the top 10 institutions listed in Table 2. Nantong University led the field with 143 papers, demonstrating outstanding research strength in the molecular mechanisms of PNI. Following closely were Sun Yat-sen University (38 papers), University of California (22 papers), Shanghai Jiao Tong University (21 papers), and Kyushu University (19 papers), forming the leading research group in the field. The institutional collaboration network shown in Figure 3D highlights a dense cooperation network within Chinese institutions, with Nantong University, Sun Yat-sen University, and Shanghai Jiao Tong University as key nodes in domestic collaborations. International collaborations were mainly concentrated between top Chinese institutions and well-known overseas universities, exemplified by the partnerships between Shanghai Jiao Tong University and Johns Hopkins University, as well as between Peking University and the University of California.

**Table 2:** Top 10 most productive institutions in research on the molecular mechanisms of peripheral nerve injury.

Rank	Institution	Publications	Country
1	Nantong University	143	China
2	Sun Yat-sen University	38	China
3	University of California	22	United States
4	Shanghai Jiao Tong University	21	China
5	Kyushu University	19	Japan
6	Fudan University	18	China
7	Johns Hopkins University School of Medicine	18	United States
8	Rutgers State University	18	United States
9	Southern Medical University	18	China
10	University of California, San Diego	18	United States

### 3.3 Analysis of authors

**Figure 4:**
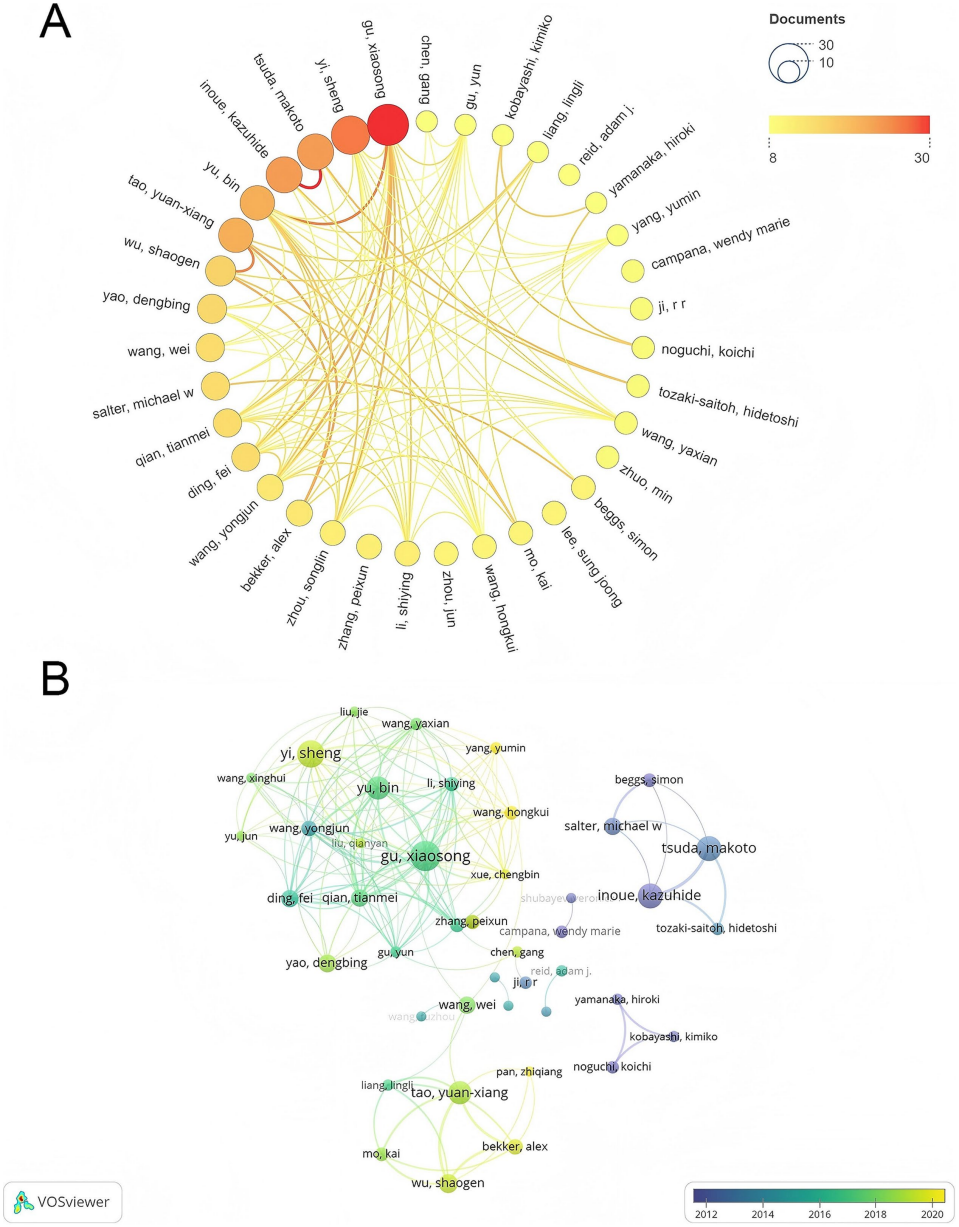
Author collaboration network in PNI molecular mechanism research. **(A)** Author collaboration network generated by VOSviewer, with nodes representing scholars. Node size is proportional to publication volume, and lines represent collaboration relationships. Line thickness reflects collaboration strength, and different colors represent distinct collaboration clusters. **(B)** Author collaboration timeline generated by VOSviewer combined with Pajek. The x-axis represents years, with nodes representing scholars. The closer the node color is to yellow, the later the year of collaboration.

From 2005 to 2025, a total of 9,138 authors contributed to the research on the molecular mechanisms of PNI, forming a pattern of core authors leading collaborative research with multi-country participation. Table 3 lists the top 12 authors by publication output in this field. Gu Xiaosong led with 30 papers, becoming a leading figure in the field, followed by Yi Sheng with 26 papers. Inoue Kazuhide and Tsuda Makoto both contributed 23 papers, ranking third. Tao Yuan-xiang and Yu Bin each published 21 papers, ranking fifth. These authors form the core group driving research in this area. In the author collaboration network (Figure 4A), node size is proportional to publication output, with lines representing collaborations. The timeline of author collaborations (Figure 4B), spanning from 2012 to 2020, shows a clear pattern of evolving collaboration over time, with nodes colored closer to yellow indicating later years. This map clearly depicts the development of core collaboration teams: a Chinese team led by Gu Xiaosong and Yi Sheng, whose collaboration frequency significantly increased after 2016, strengthening team cohesion; a Japanese team led by Inoue Kazuhide and Tsuda Makoto, with collaborations concentrated from 2014 to 2018; and a Western team led by Salter Michael W and Bekker Alex, whose international collaboration increased from 2016 to 2020, reflecting a strengthening of cross-border collaboration in recent years. Notably, while cross-border collaboration among core authors has steadily increased, long-term deep collaborations remain limited, with the overall density of international cooperation lower than that seen in domestic teams, suggesting further room for enhanced collaborative research.

**Table 3:** Top 12 most productive authors in research on molecular mechanisms of peripheral nerve injury.

Rank	Author	Publications	Country
1	Gu, Xiaosong	30	China
2	Yi, Sheng	26	China
3	Inoue, Kazuhide	23	Japan
4	Tsuda, Makoto	23	Japan
5	Tao, Yuan-xiang	21	China
6	Yu, Bin	21	China
7	Wu, Shaogen	16	China
8	Yao, Dengbing	15	China
9	Ding, Fei	14	China
10	Qian, Tianmei	14	China
11	Salter, Michael W	14	United States
12	Wang, Wei	14	China

### 3.4 Analysis of journals

**Figure 5:**
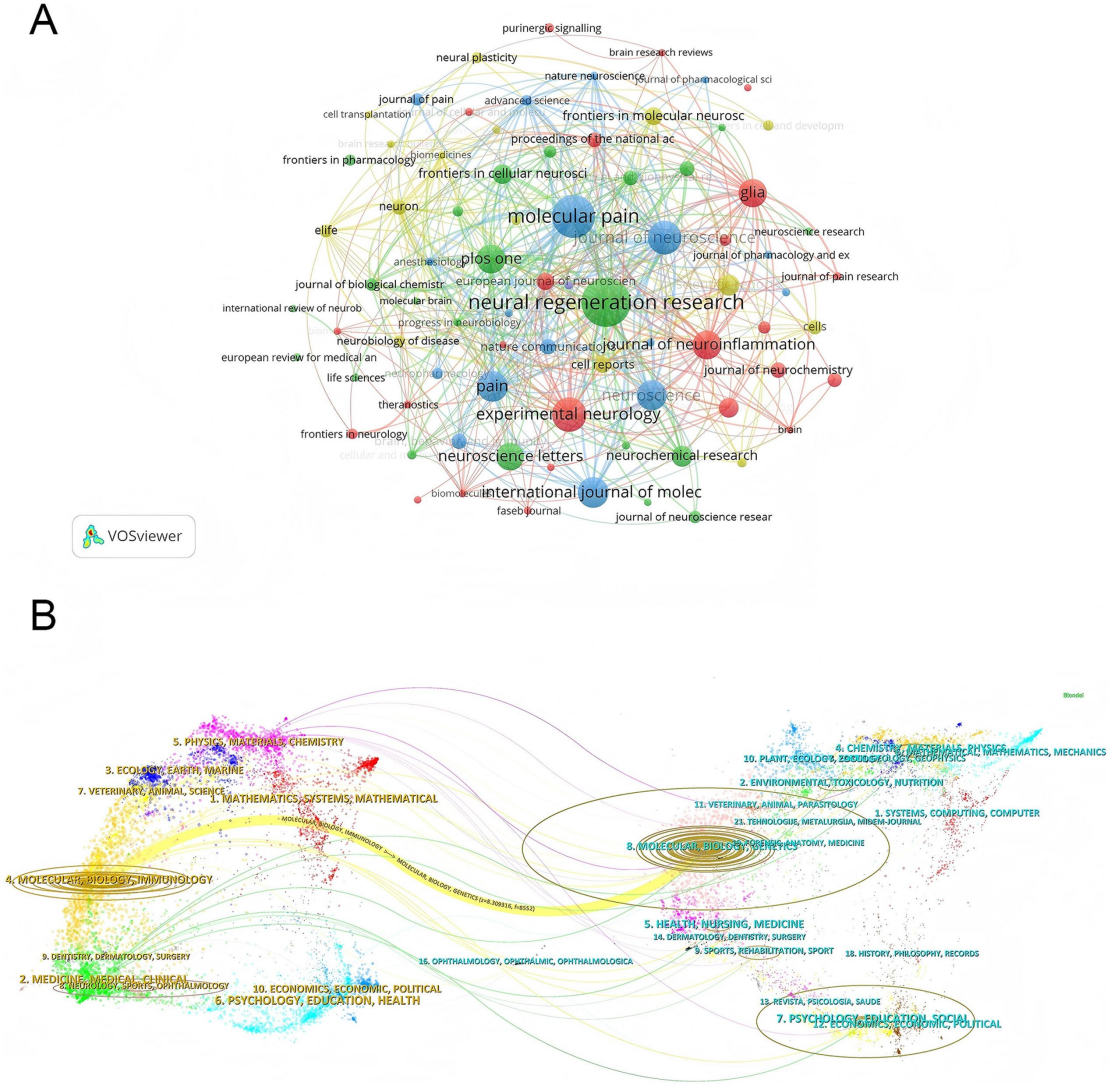
Journal collaboration network in PNI molecular mechanism research. **(A)** Journal network map generated by VOSviewer, with nodes representing journals. Node size correlates with publication volume, and lines represent thematic relationships between journals. **(B)** Journal dual-map overlay generated by CiteSpace. The left side represents the discipline clusters of citing journals (representing research frontiers), and the right side represents the discipline clusters of cited journals (representing the knowledge base). Different colors distinguish academic disciplines, and lines reflect citation relationships between disciplines.

Research in this field has been published across 566 journals, with a concentration in core journals that cover multiple disciplines. Table 4 lists the top 10 journals by publication output. *Neural Regeneration Research* ranked first with 66 papers, becoming the primary outlet for research in this field, followed by *Molecular Pain* with 55 papers. *Experimental Neurology* (41 papers), *Journal of Neuroscience* (40 papers), *International Journal of Molecular Sciences* (35 papers), and *Pain* (35 papers) ranked third through fifth. These 10 journals collectively published 401 papers, accounting for 22.3% of the total publications, forming the core journal cluster in the field. In terms of journal impact, over half of the top 10 journals are in the JCR Q1 category, with the *Journal of Neuroinflammation* having the highest impact factor (10.1), focusing on the intersection of neuroinflammation and injury repair mechanisms. Other high-impact journals include *Neural Regeneration Research* (IF = 6.7), *Pain* (IF = 5.5), and *Glia* (IF = 5.1), underscoring the academic authority of core journals in the field. The journal network map (Figure 5A) reveals five core clusters: the green cluster centered around *Neural Regeneration Research*, focusing on nerve regeneration and tissue repair; the blue cluster centered on *Molecular Pain* and *Pain*, encompassing research on pain mechanisms; and the red cluster centered on *Journal of Neuroinflammation* and *Glia*, focusing on neuroinflammation and glial cell functions. The dual-map overlay of journals (Figure 5B) reveals that the research frontier is primarily in applied fields such as Molecular Biology, Immunology and Medicine, Medical, Clinical, while the knowledge base is derived from fundamental disciplines like Molecular Biology, Genetics and Systems, Computing, Computer. The core citation path shows support from fundamental disciplines to neuro-medical applications. However, the association between the clinical disciplines and the research frontiers was relatively weak. This observation is further supported by the composition of the literature included in the present study. Among the 1,409 original research articles analyzed, only 218 were clinical studies (15.5%), whereas 1,191 were basic or animal experimental studies (84.5%). These findings indicate that research in this field is predominantly focused on basic science, while clinical translation remains comparatively limited.

**Table 4:** Top 10 most published journals in research on molecular mechanisms of peripheral nerve injury.

Rank	Journal	Publications	JCR (2024)	IF (2024)
1	Neural Regeneration Research	66	Q1	6.7
2	Molecular Pain	55	Q3	2.8
3	Experimental Neurology	41	Q1	4.2
4	Journal of Neuroscience	40	Q2	4.0
5	International Journal of Molecular Sciences	35	Q1	4.9
6	Pain	35	Q1	5.5
7	Neuroscience	34	Q3	2.8
8	Journal of Neuroinflammation	32	Q1	10.1
9	Plos One	32	Q2	2.6
10	Glia	31	Q1	5.1

### 3.5 Analysis of keywords

Keywords are central to identifying the core themes of research articles. By analyzing the frequency co-occurrence relationships and burst detection of keywords we can precisely identify research hotspots and trends in the field (12). In this study VOSviewer software was used to visualize keyword co-occurrence networks while CiteSpace was utilized to detect keyword bursts and Bibliometrix was used to generate quadrant plots.

**Figure 6:**
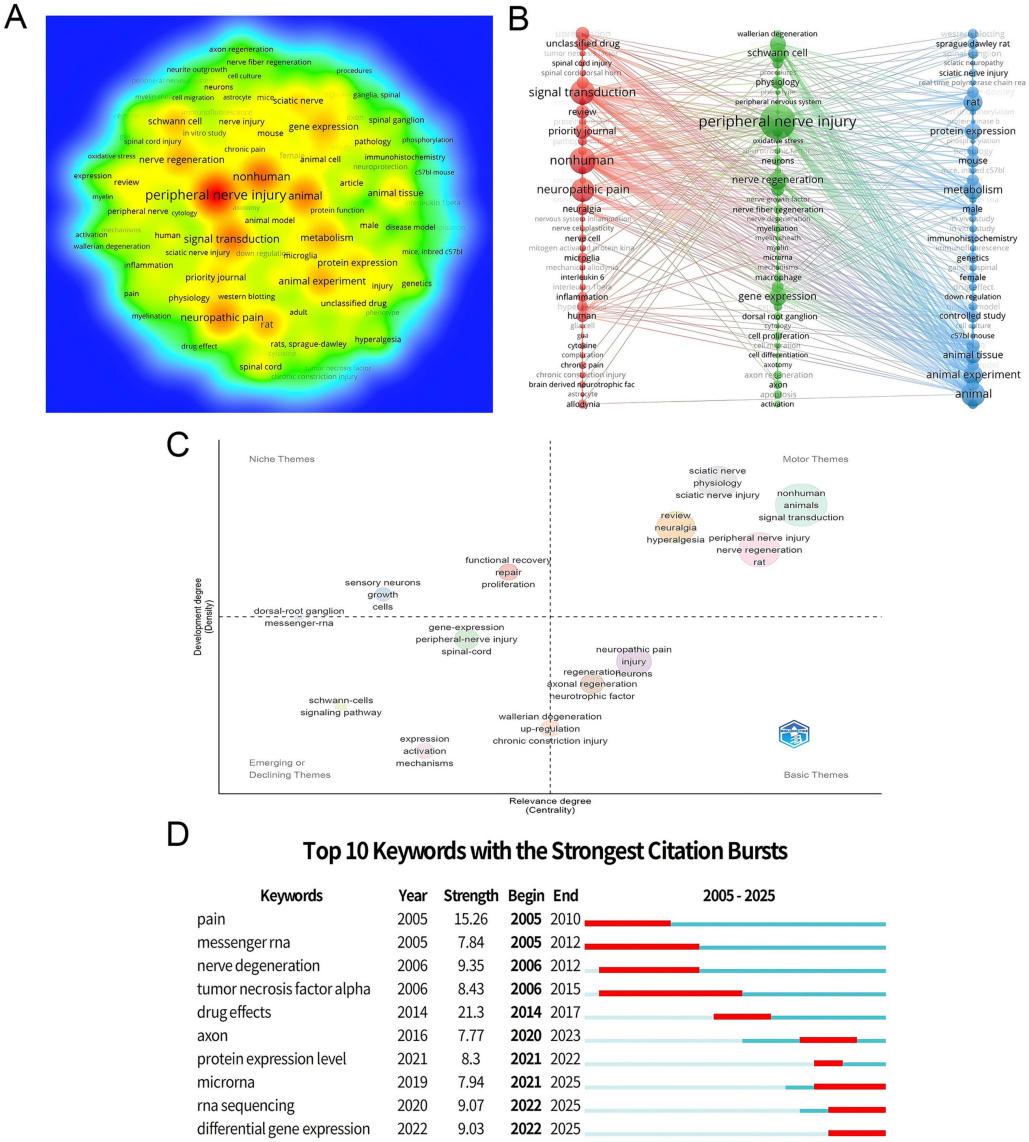
Keyword analysis in PNI molecular mechanism research. **(A)** Keyword heatmap generated by VOSviewer, with node size correlating with keyword frequency. The color distribution visually represents the concentration of keywords. **(B)** Keyword co-occurrence network generated by VOSviewer, with different colors representing different clusters. Line thickness indicates the strength of co-occurrence relationships between keywords. **(C)** Keyword quadrant map based on the “Centrality-Density” model, classifying research directions into “Basic Themes,” “Motor Themes,” “Niche Themes,” and “Emerging or Declining Themes.” **(D)** Top 10 keywords with the highest burst strength, generated by CiteSpace. “Strength” represents the attention intensity (higher values indicate greater attention), and “Begin/End” marks the periods of high burst intensity. The red section of the timeline indicates the active period, while the cyan section represents the sustained association period.

A total of 13,097 keywords were identified from 2005 to 2025, with 130 keywords occurring more than 50 times. The keyword heat map (Figure 6A) shows that “peripheral nerve injury” is the central theme in this field, with surrounding keywords such as “neuropathic pain,” “signal transduction,” and “nerve regeneration” forming dense clusters, reflecting the focus of the research. In the high-frequency keyword ranking (Table 5), “peripheral nerve injury” ranks first with 1,255 occurrences and a total association strength of 19,728, remaining the central theme in the field. “Nonhuman,” “signal transduction,” “animal,” and “neuropathic pain” all appeared more than 600 times, ranking second to fifth, highlighting the dominance of animal experiments and the focus on signal transduction and neuropathic pain as core research areas. Additionally, keywords like “nerve regeneration,” “schwann cell,” and “metabolism” appeared more than 400 times, further confirming that the mechanisms of repair and cell regulation after peripheral nerve injury are key research directions.

**Table 5:** Top 10 most frequent keywords in research on molecular mechanisms of peripheral nerve injury.

Rank	Keyword	Occurrences	Total link strength
1	Peripheral nerve injury	1,255	19,728
2	Nonhuman	787	16,990
3	Signal transduction	700	14,720
4	Animal	680	15,131
5	Neuropathic pain	642	8,411
6	Animal experiment	490	12,156
7	Rat	472	9,472
8	Metabolism	471	11,084
9	Nerve regeneration	463	8,315
10	Schwann cell	415	6,016

The keyword co-occurrence network (Figure 6B) formed three tightly connected clusters: the red cluster centered on “neuropathic pain” and “signal transduction,” linked to “inflammation” and “microglia,” focusing on signaling mechanisms related to pain and inflammation; the green cluster centered on “peripheral nerve injury,” linking “schwann cell,” “nerve regeneration,” and “gene expression,” corresponding to research on cell regulation, regeneration mechanisms, and gene expression after injury; and the blue cluster centered on “animal” and “animal experiment,” linked to “protein expression” and “metabolism,” focusing on animal models and protein metabolism. The three clusters represent a complete research trajectory from injury models and pathological manifestations to mechanism regulation.

The quadrant analysis of keywords (Figure 6C) based on the “relevance-development” model reveals the research structure, dynamics, and development potential of the field. In the “Basic Themes” quadrant, keywords such as “neuropathic pain” and “injury” have high relevance, reflecting the long-term core research on pathological phenotypes (e.g., pain) and molecular mechanisms of injury. In the “Motor Themes” quadrant, keywords like “nerve regeneration” and “signal transduction” show both high relevance and high development, indicating active research on post-injury repair mechanisms and molecular signaling networks. In the “Niche Themes” quadrant, keywords like “dorsal root ganglion “and “messenger rna “have lower development potential, suggesting that the field has entered a stage of more detailed exploration, focusing on specific tissues or molecules in injury and repair. These niche themes may become future growth points. The “Emerging or Declining Themes” quadrant, including keywords like “schwann cells” and “signaling pathway,” indicates that research on Schwann cell regulation and new signaling pathways is gaining attention and may become a new frontier in the field.

The burst analysis of keywords (Figure 6D) identified the top 10 keywords with the strongest burst intensity, reflecting the evolving research frontiers at different periods. From 2005 to 2010, “pain” emerged as a key early hotspot, while “messenger rna “also became a focus, laying the foundation for research on the “pain phenotype and molecular basis.” From 2006 onwards, keywords like “nerve degeneration” and “tumor necrosis factor alpha” continued to emerge, highlighting the in-depth exploration of injury pathology and inflammatory factors. Between 2014 and 2017, “drug effects” peaked with the highest burst intensity, reflecting an increased focus on therapeutic interventions for injury. After 2020, keywords such as “axon,” “microrna,” “RNA sequencing,” “differential gene expression,” and “protein expression level” gained prominence, with “microrna,” “RNA sequencing,” and “differential gene expression” continuing to emerge through 2025, becoming some of the most active research frontiers. This analysis demonstrates the field’s shift from “early exploration of pathological phenotypes” to a focus on “pathological mechanisms and intervention strategies,” ultimately advancing toward “molecular regulation (non-coding RNA, gene/protein expression) and technology-driven (sequencing technologies)” for precise mechanism research. The integration of “molecular network analysis and advanced experimental technologies” will be the core research trend in the future.

### 3.6 Citation analysis

We conducted citation analysis of the 1,799 papers included in this study using VOSviewer software to identify high-impact papers in the field of molecular mechanisms of PNI. This analysis provides insights into research hotspots and trends. Table 6 lists the top 10 most-cited papers, which include 2 original research articles and 8 reviews. Latremoliere’s 2009 study, published in *J Pain*, ranks first with 3,001 total citations. It focuses on the mechanisms of central sensitization and pain hypersensitivity, serving as a key paper that connects various research directions in the field. The 2005 study by the Coull team, published in *Nature* (1,709 citations), and the 2021 review by Finnerup, published in *Physiol Rev* (986 citations), rank second and third, respectively. The former reveals the role of BDNF (brain-derived neurotrophic factor) derived from microglia in the regulation of neuropathic pain after PNI, while the latter systematically reviews the mechanisms and treatments of neuropathic pain, providing an important theoretical foundation for subsequent studies. Notably, a 2018 review by Inoue’s team, published in *Nat Rev Neurosci* (680 citations), focuses on the cellular and molecular mechanisms of microglia in neuropathic pain and highlights the “neuroimmune interaction regulation” as a current research hotspot. This paper suggests that “targeting glial cells for therapeutic interventions” will become an important direction for future translational research.

**Table 6:** Top 10 most cited papers in research on molecular mechanisms of peripheral nerve injury.

Rank	Author	Journal	Year	Title	Total citations
1	Latremoliere	J Pain	2009	Central sensitization: a generator of pain hypersensitivity by central neural plasticity	3,001
2	Coull	Nature	2005	BDNF from microglia causes the shift in neuronal anion gradient underlying neuropathic pain	1,709
3	Finnerup N	Physiol Rev	2021	Neuropathic pain: from mechanisms to treatment	986
4	Chen	Nat Rev. Drug Discov	2013	Adenosine receptors as drug targets--what are the challenges?	779
5	Tsuda	Trends Neurosci	2005	Neuropathic pain and spinal microglia: a big problem from molecules in “small” glia	728
6	Navarro X	Prog Neurobiol	2007	Neural plasticity after peripheral nerve injury and regeneration	699
7	Ren	Nat Med	2010	Interactions between the immune and nervous systems in pain	699
8	Inoue	Nat Rev. Neurosci	2018	Microglia in neuropathic pain: cellular and molecular mechanisms and therapeutic potential	680
9	Feldman E	Neuron	2017	New horizons in diabetic neuropathy: mechanisms, bioenergetics, and pain	664
10	Scheib	Nat Rev. Neurol	2013	Advances in peripheral nerve regeneration	620

**Figure 7:**
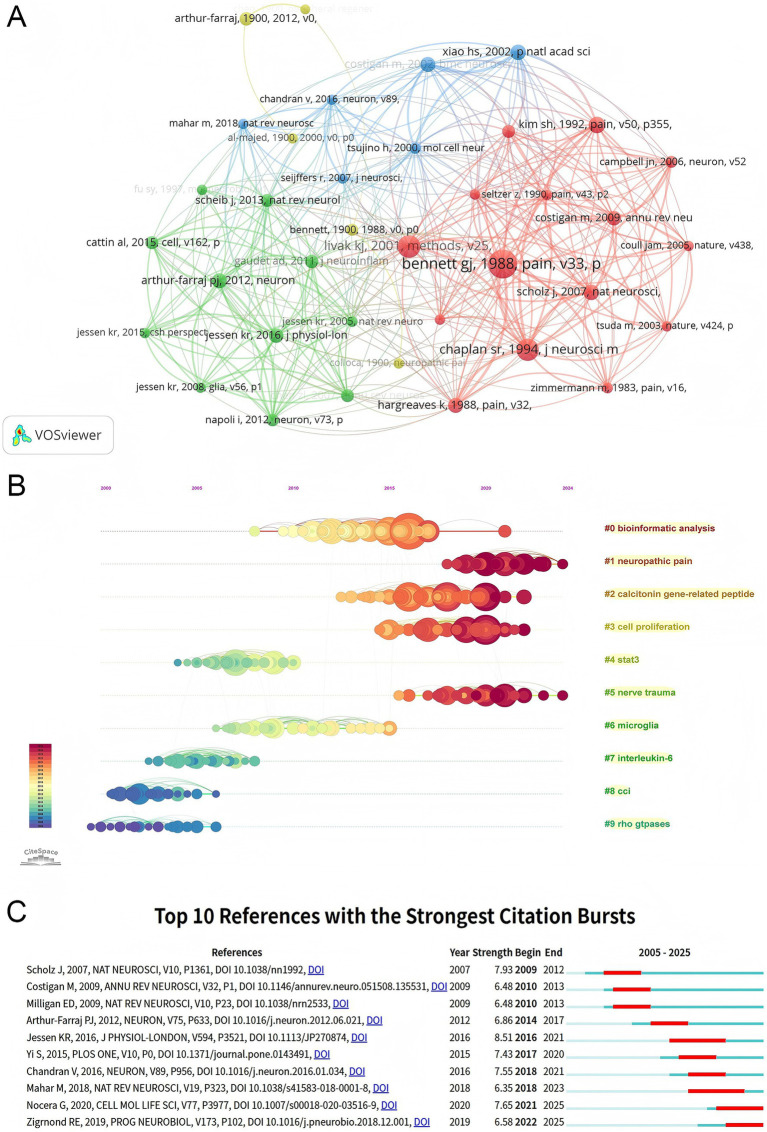
Co-citation analysis of references in PNI molecular mechanism research. **(A)** Co-citation network generated by VOSviewer, with nodes representing references. Node size correlates with citation frequency, and line thickness reflects the co-citation strength between references. **(B)** Core cluster timeline map generated by CiteSpace, with the x-axis representing time. Node position corresponds to the citation time of the reference, and node size correlates with citation frequency. Different colors represent different clusters. **(C)** Top 10 references with the strongest citation bursts, generated by CiteSpace. The burst intensity reflects the level of attention a reference received during a specific period, with the “Begin” and “End” labels marking the duration of the high burst period.

The literature included in this study cites a total of 40,138 references, of which 37 papers were cited more than 25 times. Using VOSviewer, we generated a co-citation network of these highly cited references (Figure 7A). In this network, the size of the nodes is positively correlated with citation frequency, and the strength of the connecting lines indicates the degree of co-citation. The network is divided into different clusters based on citation relationships. The red cluster, centered around “Bennett GI, 1988, *Pain*” and “Coull JAM, 2005, *Nature*,” reflects a concentrated research foundation in the field of neuropathic pain. The green cluster, based on the works of Jessen KR (2005, 2008, 2016), shows a continuous body of research focusing on Schwann cell-related studies. The blue cluster, which includes the work of Chandran V (2016, *Neuron*), indicates a broader network of connections with other research directions in the field.

The cluster theme analysis (Figure 7B) reveals that the co-citation network can be grouped into 10 core clusters. The nodes in the figure are positioned along the horizontal time axis, indicating when the references were cited. The size of the nodes correlates with citation frequency, presenting clear trends and shifts in research themes over time. From 2005 to 2010, research hotspots focused on fundamental mechanisms such as “neuropathic pain” (#1) and “Rho GTPases” (#9). From 2010 to 2018, inflammation-related topics, such as “microglia” (#6) and “interleukin-6” (#7), gradually emerged as central themes. After 2018, topics such as “bioinformatics analysis” (#0) and “STAT3 signaling” (#4) became more prominent, indicating the field’s transition toward multidisciplinary and precise regulatory mechanisms.

Burst detection analysis (Figure 7C) highlighted the top 10 keywords with the strongest burst intensity, all with values exceeding 6.3. The study by Jessen KR (2016, *J Physiol London*) exhibited the highest burst intensity of 8.51, from 2016 to 2021, focusing on Schwann cell regulation in nerve regeneration. Nocera G’s 2020 study (*Cell Mol Life Sci*) had a burst intensity of 7.65, continuing through 2025, while Zigrnond RE’s 2019 paper (*Prog Neurobiol*) had a burst intensity of 6.58. These papers, focusing on the molecular repair mechanisms following nerve injury and macrophage-mediated inflammation regulation, confirm the trends revealed by the clustering timeline. Additionally, the landmark 2012 paper by Arthur-Farraj PJ (*Neuron*) with a burst intensity of 6.86 (2014–2017) laid the foundation for research into molecular mechanisms of nerve regeneration.

## 4 Discussion

### 4.1 Summary of basic information

This study conducted a comprehensive bibliometric analysis of research on the molecular mechanisms of PNI from 2005 to 2025. Through the retrieval and analysis of 1,799 relevant publications, we identified the long-term development trends and current research hotspots in this field. First, we found that research on PNI has grown steadily over the past two decades, with a particularly significant increase in publication output in the past 10 years, peaking at 134 publications in 2025. This growth trend indicates that PNI, as an important area of biomedical research, is receiving increasing attention, with both the depth and breadth of research expanding continuously.

Geographically, research on the molecular mechanisms of PNI is mainly concentrated in China, the United States, and Japan. In particular, China has seen rapid growth in the number of publications in recent years, with 679 papers published, making it one of the leading contributors in this field. The collaborative networks of research institutions also show a clustering effect, with close cooperation between top institutions within China, such as Nantong University and Sun Yat-sen University, playing a key role in both domestic and international research collaboration. However, further international institutional cooperation is needed to promote the development of this field.

Analysis of authors reveals that scholars such as Gu Xiaosong (30 papers) and Yi Sheng (26 papers) occupy central positions, particularly in China, where they have formed stable research teams since 2012. Additionally, Japanese scholars like Inoue Kazuhide and Tsuda Makoto have also had a profound impact on the field. Journal analysis shows that *Neural Regeneration Research* and *Molecular Pain* are key platforms for publishing research in this area, with most journals belonging to the JCR Q1 category, reflecting the interdisciplinary nature of PNI research. However, the dual-map overlay of journals reveals a gap between basic research outcomes and clinical applications.

### 4.2 Research hotspots and trends

By visualizing high-citation papers, keywords, and co-cited literature, we identified several key research hotspots in the molecular mechanisms of PNI, primarily focusing on nerve regeneration, Schwann cell function, neuropathic pain, inflammation regulation, and molecular signaling pathways. Emerging bioinformatics technologies such as high-throughput genomics, proteomics, transcriptomics, and single-cell sequencing have refined the molecular pathway regulation and are expected to drive future trends in PNI research.

#### 4.2.1 Schwann cells and nerve regeneration

Schwann cells play a central role in the repair of PNI and have become a primary research focus in this field. Keyword frequency analysis revealed that “schwann cell” appeared more than 400 times, indicating its prominence as a major research focus in this field. In the keyword strategic diagram (four-quadrant analysis), “schwann-cells” was located in the Emerging or Declining Themes quadrant, suggesting that although its thematic centrality remains to be strengthened, its degree of development is increasing. This pattern indicates a growing research interest in the functional regulation of Schwann cells. Consistently, citation burst analysis identified a landmark study published by Jessen KR in 2016, which focused on the regulatory role of Schwann cells in nerve regeneration and exhibited a strong citation burst intensity of 8.51, highlighting its pivotal impact within the field. Recent studies have increasingly concentrated on the function and molecular mechanisms of Schwann cells, particularly their role in axonal regeneration and remyelination (13). For example, Xu et al. demonstrated that neurotrophin-3 (NT-3) promotes axonal regeneration following peripheral nerve injury by maintaining the repair phenotype of Schwann cells through activation of the TrkC/ERK/c-Jun signaling pathway (14). Recently, researchers have successfully activated the regenerative potential of Schwann cells through gene editing technologies, promoting nerve repair after injury (15). Future studies may focus on exploring how Schwann cells interact with other cell types, such as macrophages, during nerve regeneration to enhance repair outcomes.

#### 4.2.2 Neuropathic pain and inflammatory response

Neuropathic pain, a common complication following peripheral nerve injury, involves complex mechanisms that are not yet effectively treated. The bibliometric results of this study strongly confirm its position as a core research focus in the field. Keyword frequency analysis revealed that “neuropathic pain” appeared over 600 times, ranking as the fifth most frequent keyword. In the keyword strategic diagram (four-quadrant analysis), “neuropathic pain” was positioned in the Basic Themes quadrant, indicating its high association and solidifying it as a long-standing core research foundation in the field. In recent years, researchers have increasingly recognized the critical role of microglia, macrophages, and neuroinflammatory responses in the onset and progression of neuropathic pain (16, 17). This finding is in strong agreement with the “neuropathic pain-inflammation-microglia” association pattern presented in the keyword co-occurrence network’s red cluster. Studies have shown that activation of microglia and the secretion of cytokines (e.g., TNF-α, IL-1β) enhance pain responses and influence the persistence of pain by modulating neuronal plasticity after nerve injury (18). Temporal clustering analysis of the literature also suggested that, from 2010 to 2018, the focus of research gradually shifted toward key neuroinflammatory targets, such as microglia and interleukin-6.

Furthermore, as the understanding of inflammatory mechanisms deepens, more research is focusing on neuroimmune interactions, such as how Schwann cells and microglia cooperate in regulating the development of neuropathic pain after nerve injury (19). A study by Gu et al. demonstrated that, after peripheral nerve injury, early inflammatory responses facilitate the clearance of debris and promote axonal regeneration and myelin reconstruction; however, if inflammation is prolonged or excessively activated, it may lead to neural tissue damage and inhibit regenerative processes (20). Therefore, the precise regulation of the intensity and duration of post-injury inflammatory responses has emerged as one of the key research hotspots in the field of PNI.

#### 4.2.3 MicroRNAs and nerve regeneration

With the application of RNA sequencing technologies, non-coding RNAs, especially microRNAs (miRNAs), have gained increasing attention in PNI research. Keyword burst analysis revealed a pronounced surge in the term “microrna” after 2020, with a burst strength exceeding 7.8 and a burst duration extending through 2025, identifying it as one of the most active current research frontiers. Concurrently, related terms such as “RNA sequencing” also exhibited strong citation bursts, indicating that the integration of miRNA research with high-throughput sequencing technologies has become a core developmental direction in this field. miRNAs regulate gene expression and play a vital role in nerve injury and repair processes. Studies have shown that specific miRNAs (e.g., miR-21, miR-155, miR-133b) play key roles in Schwann cell phenotypic transformation, axonal regeneration, and inflammatory responses after PNI. These miRNAs target multiple key genes to regulate the cell cycle, apoptosis, and immune responses, thereby affecting nerve repair after injury (21–23).

Furthermore, miRNAs not only influence Schwann cell function to promote nerve regeneration but also modulate pain responses and neuroinflammation, providing new molecular targets for PNI treatment (24). This observation is consistent with the positioning of “messenger-rna” in the keyword strategic diagram (four-quadrant analysis), where it appears as a specialized subtheme reflecting increasingly refined and focused exploration within the field. Collectively, these results underscore a clear trend toward in-depth investigation of specific molecular regulatory mechanisms in PNI research. It is also worth noting that long non-coding RNAs (lncRNAs) have gradually attracted attention in PNI. Studies have shown that lncRNAs interact with miRNAs and transcription factors to regulate the expression of genes related to nerve repair (25). Thus, the combined regulation of miRNAs and lncRNAs may become an emerging strategy for PNI treatment.

#### 4.2.4 Molecular signaling and gene expression regulation

Molecular signaling pathways have been extensively studied for their roles in PNI repair. Keyword frequency analysis revealed that “signal transduction” appeared more than 600 times, ranking as the third most frequent keyword. In the keyword strategic diagram (four-quadrant analysis), “signal transduction” was positioned in the Motor Themes quadrant, demonstrating both high centrality and high developmental degree, thereby indicating its status as an active and driving research hotspot. Pathways such as c-Jun and PI3K/AKT play significant roles in nerve repair. Notably, c-Jun is a central factor in axonal regeneration after nerve injury, promoting Schwann cell dedifferentiation and proliferation (26). Additionally, the role of the STAT3 signaling pathway in nerve regeneration is also critical. Activation of STAT3 promotes the regeneration of damaged nerves while inhibiting apoptotic responses, making it a vital therapeutic target for nerve repair (27). These findings are consistent with citation clustering results, in which “STAT3 signaling regulation” emerged as a newly developing research hotspot.

With the advancement of gene editing technologies, an increasing number of studies have begun to explore the use of tools such as CRISPR-Cas9 to regulate key genes in order to enhance cellular repair capacity. For example, Hsu et al. developed a CRISPR activation (CRISPRa) system that simultaneously upregulated the expression of neurotrophic factors—BDNF, GDNF, and NGF—in adipose-derived stem cells in a rat sciatic nerve injury model (28). This approach significantly enhanced axonal regeneration, myelin reconstruction, and functional recovery. Future research may further investigate how to integrate these signaling pathways with gene regulation strategies to develop more effective therapeutic approaches for PNI.

#### 4.2.5 Emerging bioinformatics technologies

With the rapid development of high-throughput genomics, proteomics, transcriptomics, and single-cell sequencing technologies, PNI research has shifted from basic mechanistic studies to precision medicine. This trend was particularly evident in the present bibliometric analysis. Keyword burst analysis revealed that, after 2020, technology-related terms such as “RNA sequencing” “differential gene expression” and “protein expression level” exhibited pronounced bursts that persisted through 2025. Furthermore, co-citation clustering analysis demonstrated a marked increase in node density for the theme of “bioinformatics analysis” after 2018, identifying it as an emerging research hotspot in the field. These technologies have provided strong support for identifying changes in gene expression, key pathways, and molecular mechanisms following nerve injury, helping researchers discover more potential therapeutic targets. For instance, Weiss et al. employed RNA sequencing to analyze gene expression changes in rat sciatic nerve and muscle tissues following polyethylene glycol (PEG)-enhanced primary neurorrhaphy (29). Their study identified a series of significantly differentially expressed genes and associated pathways closely related to nerve development and growth, contributing to a deeper understanding of nerve repair mechanisms and potential therapeutic targets.

In addition, a quantitative mass spectrometry–based proteomic analysis of proximal and distal segments of human digital nerves after injury identified a total of 3,914 proteins, among which 127 exhibited significant differential expression between the two segments. Enrichment analysis indicated that upregulated proteins in the distal nerve segment were primarily involved in cellular stress responses, neuromuscular junction stability, axon guidance, and angiogenesis, whereas downregulated proteins were associated with synaptic transmission, autophagy, and cell adhesion (30). These findings provide important insights into the mechanisms underlying nerve injury and repair and offer valuable clues for the identification of potential therapeutic targets. Collectively, these emerging bioinformatics technologies have opened new avenues for PNI treatment and hold promise for the development of more precise and personalized therapeutic strategies.

### 4.3 Strengths and limitations

The core strength of this study lies in its systematic analysis of 1,799 publications from 2005 to 2025, leveraging a large sample size and long-term time-series data, coupled with multi-software, multi-dimensional visualization techniques, to provide a comprehensive overview of the PNI field. Through this systematic approach, we not only update the research paradigm in this domain but also uncover emerging research directions and technological applications. Particularly, in the context of the accelerating application of new technologies such as RNA-seq, single-cell sequencing, and proteomics, this study enables the identification of subtle changes and emerging trends at a macro level, offering new perspectives and insights into the forefront of PNI research.

This study also has some limitations. First, only English-language literature was included to meet the requirements of CiteSpace software, which may have excluded research from non-English-speaking countries, thus limiting the perspective and data of the study. Second, potential bias may be introduced by the search strategy. General terms such as “molecular mechanism” and “signaling pathway” were used, which may have excluded relevant mechanistic studies that did not use these terms in their titles or abstracts. Finally, while bibliometric methods provide valuable quantitative data, they do not allow for an in-depth analysis of the quality, methodology, or clinical application outcomes of individual studies.

## 5 Conclusion

This study has revealed several key research hotspots in the molecular mechanisms of PNI, including Schwann cells, neuropathic pain, inflammation responses, non-coding RNAs, and molecular signaling pathways. These research hotspots have not only deepened our understanding of the basic mechanisms of PNI but also provided new directions for clinical treatment. Furthermore, the study highlights the potential impact of advances in high-throughput genomics, proteomics, transcriptomics, and single-cell sequencing technologies on the molecular mechanisms of PNI. With the development of precision medicine and gene editing technologies, future research is expected to focus on exploring these emerging therapeutic strategies and achieving more effective nerve repair and functional recovery.

## Data Availability

The original contributions presented in the study are included in the article/Supplementary material, further inquiries can be directed to the corresponding authors.
