# Supplementary material for: Trends in peripheral nerve injury research: a bibliometric analysis focused on molecular mechanisms
Source: Front Neurol. 2026 Mar 6;17:1771375. doi: 10.3389/fneur.2026.1771375 (PMC13003782; doi:10.3389/fneur.2026.1771375)
Supplement: Supplementary file 1 [file Table_1.docx]

Supplementary Table 1. Merging and modification records of country/region names and keywords.

| Standardization | Label | Replace by |
| --- | --- | --- |
| Country/Region | United States | USA |
|  | Peoples R China | China |
|  | England | United Kingdom |
|  | Scotland | United Kingdom |
|  | Wales | United Kingdom |
|  | Turkiye | Turkey |
|  | Taiwan | China |
|  | Hong Kong | China |
|  | Macau | China |
| Keyword | axonal regeneration | axon regeneration |
|  | gene expression regulation | gene expression |
|  | Cells cultured | cell culture |
|  | dorsal-root ganglion | dorsal root ganglion |
|  | gene-expression | gene expression |
|  | peripheral-nerve injury | Peripheral nerve injury |
|  | schwann-cells | schwann cell |
|  | sciatic-nerve | sciatic nerve |
|  | spinal-cord | spinal cord |
|  | up-regulation | upregulation |
|  | animals | animal |
|  | axons | axon |
|  | peripheral nerves | peripheral nerve |
|  | macrophages | macrophage |
|  | schwann cells | schwann cell |
|  | cytokines | cytokine |
|  | disease models | disease model |
|  | humans | human |
|  | rats | rat |
